# Oxidative stress – Alzheimer’s disease – DNA methylation: the role of arsenic

**DOI:** 10.1042/EBC20253019

**Published:** 2025-07-01

**Authors:** Daniele Antinori, Marco Lucarelli, Andrea Fuso

**Affiliations:** 1 Department of Experimental Medicine, Sapienza University of Rome, Rome, Italy; 2 Pasteur Institute Cenci Bolognetti Foundation, Sapienza University of Rome, Rome, Italy; 3 CRiN, Center for Research in Neurobiology D. Bovet, Sapienza University of Rome, Rome, Italy

**Keywords:** arsenic, Alzheimer’s disease, DNA methylation, epigenetics, neurodegeneration, oxidation

## Abstract

Alzheimer’s disease (AD) is a neurodegenerative disease, representing the seventh cause of death worldwide and the first cause of dementia. Several pathogenic mechanisms have been connected to this pathology, including protein aggregation, oxidative stress, metabolic dysfunction, mitochondrial dysfunction, neuroinflammation, synaptic dysfunction, and cell death. The etiology of AD is multifactorial, suggesting that, in addition to a genetic component, the environment may strongly influence its onset and progression. Exposure to heavy metals, such as lead, cadmium, mercury, and arsenic (As), is known to be associated with AD, with As showing one of the strongest correlations, in relation to the epigenetic changes. The World Health Organization (WHO) set a very low limit for its concentration to 10 μg/l in drinking water. The possibility that As may induce epigenetic effects is a recent hypothesis. Evidence, so far, suggests that As may induce DNA hypomethylation in the brain, by mechanisms not yet completely disclosed. This minireview aims to provide evidence to support the role of As exposure in AD, maintaining a focus on oxidative stress and ferroptosis, with a perspective on DNA methylation.

## Introduction

Alzheimer’s disease (AD) is one of the most concerning diseases of our times, being the seventh leading cause of death, globally. According to the World Health Organization (WHO), more than 55 million people have dementia worldwide, with 10 million new cases every year. AD represents 60–70% of cases [1].

To date, AD remains a pathology yet to be fully understood, although years of research have been dedicated to the comprehension of the causes of the disease. Hallmarks of this illness are various and intertwined. One of the most relatable features is the presence of specific protein aggregates that contribute to the pathology: amyloid plaques and neurofibrillary tangles. Amyloid plaques are extracellular aggregates made of amyloid-β (Aβ) peptide, which derives from the alternative amyloidogenic processing of the amyloid precursor protein (APP), by the β-secretase [encoded by beta amyloid cleaving enzyme 1 (*BACE1*)], and the γ-secretase [whose catalytic subunit is encoded by presenilin 1 (*PSEN1*)]. Neurofibrillary tangles are constituted by the hyperphosphorylated tau protein. Tau is a microtubule-associated protein, and in an AD context, its hyperphosphorylation brings to its detachment from microtubules and aggregation, causing neurite destruction and cell death [2]. Other associated molecular mechanisms, characteristic of AD, are related to oxidative stress [3], metabolic dysfunction [4], mitochondrial dysfunction [5], neuroinflammation [6], synaptic dysfunction [7], and cell death [8].

The exact etiology of the disease has not yet been elucidated, but some risk factors have been unraveled. Risk for AD and related dementias is attributable to both genetic and environmental factors [9].

Allelic variants of some genes involved in amyloidogenesis, like *APP, PSEN1, and PSEN2,* have a penetrance of 100% for this pathology [10]. Other minor pathogenetic gene variants include *APOE, SORL1*, *BIN1*, *PICALM*, CD2AP*, CLU, PTK2B*, *TREM2*, and others, with more to be discovered [11].

The presence of some diseases can also predispose to the insurgence of AD. They include type 2 diabetes, cardiovascular diseases, depression, and gastrointestinal diseases [12].

Environmental risk factors have been recognized to have a significant role in the development of AD. Among them, infections, diet, air pollution, and metal exposure are primarily considered [13].

Many environmental chemicals are well known to be neurotoxic or involved in the onset and progress of neurodegenerative disorders [14]. The human brain is particularly susceptible to the toxic effects of heavy metals, such as lead, cadmium, mercury, and arsenic (As). It has been proved that these compounds can damage the central nervous system and lead to cognitive and behavioral impairments [15,16].

Wei et al. analyzed the blood content of patients with cognitive impairment and found that As has the strongest correlation with the neurological state, when considering the epigenetic effects of these metals [17].

The present review is focused specifically on analyzing the effects of As exposure in AD.

### Arsenic

As is a ubiquitous metalloid element and is one of the few that can be metabolized inside the human body [18]. It exists in three valence states, As0 (metalloid), AsIII (arsenite), and AsV (arsenate), and as arsine gas, having the formula AsH3. In water, it is mainly found in the form of arsenite and arsenate ion, while in the Earth’s crust, it is often conjoined with sulfur, mostly as arsenopyrite (FeAsS), or with other metals [19]. It can also be found in the air, coupled with oxygen in the form of arsenic trioxide (As₂O₃) [15,20]. Drinking water is the main source of As contamination, followed by food ingestion and air breathing [21]. WHO indicated 10 μg/l as the limit concentration value of As in drinking water [22].

Shaji et al. reported that groundwater of 107 countries goes beyond WHO limit. Furthermore, As concentration in water bodies, food, and soils in the world is increasing with time [20]. Except for natural disasters, As concentration in soil and groundwater can vary dependently on human activities, such as industries, agriculture, and sewage [23,24].

### Arsenic metabolism

As^III^ and As^V^ are defined as inorganic As (iAs), and they represent the principal toxic contaminant form. However, when taken into the body, they undergo a metabolic processing, forming various adducts [16]. The principal hypothesis of how As is processed is described below. Until 2004, the in-force hypothesis suggested that the trivalent forms of As [As^III^ and monomethylarsonous acid (MMA^III^)] are directly methylated, forming the pentavalent methylated analogs monomethylarsonic acid (MMA^V^) and dimethylarsinic acid (DMA^V^) [25,26]. The passage from the pentavalent status of various As forms to the trivalent state is mediated by a glutathione (GSH)-dependent reaction (As^V^ to As^III^, MMA^V^ to MMA^III^, or DMA^V^ to DMA^III^). Another hypothesis, formulated in 2004 by Hayakawa et al. to explain As metabolism,, points out that methyltransferases are more prone to add methyl groups to glutathionated forms of As: As^III^(GSH)_3_ and MMA^III^(GSH)_2_, forming MMA^III^(GSH)_2_ and DMA^III^(GSH), respectively. MMA^III^ and DMA^III^ dissociate from GSH and are then oxidized to the pentavalent analogs [27]. The methylation process of As is catalyzed by the As^III^-methyltransferase (As_3_MT), which is involved both in the reduction and in the methylation reactions [25]. AS_3_MT metabolizes As principally in the liver, but it is ubiquitously expressed, with significant expression found in the brain as well. In the past century, it was believed that As metabolism was a detoxification pathway [25]. With time, it has been discovered that some methylated forms of As are more toxic than iAs itself, making this process more intriguing. Experiments focused on the evaluation of the LD50 determined that the most toxic forms are MMA^III^ and DMA^III^ [16]. Yang et al. reported that low urinary concentration of dimethylarsenic species (DMA) and high concentration of iAs are associated with AD [28]. Even though some metabolites of As are more toxic than iAs itself, according to the Challenger’s hypothesis, but not to Hayakawa’s one, passing through the more toxic As species appears to be an obligatory step in its final excretion, as DMA^V^ is the main As form found in urine [26–29].

### Arsenic access to the brain

It has been evidenced that As and its metabolites are able to accumulate in several organs of the human body, manifesting their toxicity [18]. The kidney and liver result as the primary deposition sites. The concentration found in the brain is not comparable with that found in the two organs mentioned above, but it remains significant, nonetheless [30]. The high concentration of As deposited in the brain can be explained by the property of As to disrupt and pass through the brain blood barrier (BBB) [31]. Indeed, several works report that As exposure induces damage to the BBB, with a decrease in tight junction. Yan et al., moreover, associated the damage to a T lymphocyte response [32], while Lv et al. demonstrated an increase in serum and cortical metalloproteases MMP-2 and MMP-9 concentrations [33]. In fact, it has been described that gestational and perinatal exposure to As can be particularly neurotoxic in an already immature BBB [34]. Li et al. [31], in particular, studied As accumulation in the brain. They found that in mice, after a single oral administration of sodium arsenite (NaAsO_2_), As in the hippocampus was particularly abundant. They also reported the presence of As in the cortex, but its concentration was lower than that assessed in the hippocampus [31].

### Arsenic toxicity in AD

As exposure has been reported to be a risk factor for the impairment of the cognitive development in both early life and adulthood, showing a dose–response relationship [34,35]. The cholinergic system is fundamental for several brain functions. They include attention, learning, memory, stress response, wakefulness and sleep, and sensory information. The specific degeneration of this system is manifested in many forms of dementia. Loss of cholinergic neurons, along with dopaminergic ones, is considered the basis for AD-related psychiatric symptoms [36]. To evaluate the effect of As on cholinergic neurons, Zarazúa et al. [37] treated cholinergic SN56.B5.G4 cells and primary neuronal cells (derived from transgenic Tg2576 mice overexpressing human APPswe) with 5 or 10 μM of sodium arsenite or MMA^III^. In all cases, they assisted in a reduction in cells’ viability, meaning that, at least in these models, As can be associated with AD-like degeneration. Moreover, they analyzed the influence of As on APP processing and reported that DMA^V^ induced Aβ overproduction, while sodium arsenite decreased amyloid processing [37].

Also, the same group tested the effects of 3 ppm of sodium arsenite in Wistar rats, finding that it increased APP expression, Aβ production, and BACE-1 activity. They also reported the increase in Receptor for Advanced Glycation End-products (RAGE) expression, resulting in increased Aβ transportation through the BBB [38]. Escudero-Lourdes et al. demonstrated that treating astrocytes with 50, 125, and 500 nM MMA^III^ led to augmented expression of APP and BACE-1, as well as pro-inflammatory acute cytokines, such as Interleukin-1β (IL-1β), Interleukin-6 (IL-6), and Tumor Necrosis Factor-α (TNF-α) [39]. Wisessaowapak et al. [40] exposed differentiated SH-SY5Y neuroblastoma cells to 1–10 μM of sodium arsenate, inducing increased tau hyperphosphorylation. They also demonstrated that this process was dependent on GSK3β–ERK1/2 phosphorylation [38,40]. In Chinese hamster ovary T40 cells, Giasson et al. coherently found the hyperphosphorylation of tau after acute 500 μM sodium arsenite, but it resulted independent from changes in the activity of its principal kinases: SAPK-1, SAPK-2, ERK-1/2, GSK-3, MARK, or CDK5[41]. As has been shown to disrupt the cytoskeletal structure of neurons, causing the destruction of microtubular polymerization [42]. 3xTgAD, harboring a mutant APP (KM670/671 NL), a human mutant PS1 (M146V0) knock-in, and tau (P301L) transgenes, was treated with 3 ppm sodium arsenite. They found an increase in the hippocampus and cortex of APP and Aβ regarding amyloidogenesis and hyperphosphorylated tau for neurofibrillogenesis [43].

Very few studies assessed the effects of As exposure on cognition. One interesting study in APP/PSEN1 mice reports an association between As exposure, genome-wide histone methylation, senile plaque spreading, and cognitive deficits, showing that aAs may exacerbate the AD-like phenotype in animal models of AD [44]. The association between As and cognitive deficits in humans is still scarcely explored and may suffer from confounding factors. One paper, for example, reported a positive correlation between As serum levels and cognition, concluding that the As presence could be due to the highest consumption of fish that is also related to the assumption of docosahexaenoic acid, retaining a putative delaying effect on cognitive impairment [45].

Besides its contribution to the formation of amyloid plaques and neurofibrillary tangles, there are other mechanisms through which As can impact neurodevelopment. As can induce immediate neurotoxic effects by interfering with synaptic transmission. For example, it can reduce the activity of acetylcholinesterase. Moreover, it can induce mitochondrial dysfunction, oxidative stress and inflammation, apoptosis [35], ferroptosis [46], and epigenetic alterations [47].

### Arsenic and oxidative stress in AD

Oxidative stress is a phenomenon caused by an imbalance, in cells and tissues, between the production and accumulation of reactive oxygen species (ROS) and the ability of biological systems to detoxify these reactive products [48]. The brain is considered the organ the most prone to suffer from oxidative stress, and this is due to both structural and functional characteristics. In fact, the brain is rich in peroxidable lipids, and it is very exposed to oxygen because of its high metabolic activity. It alone consumes 20% of the breathing-derived oxygen. Moreover, cells in the brain are long-lasting, and there is low cell renewal, making them sensitive to accumulate oxidative damage. Oxidative damage happens physiologically during aging, but high oxidation levels or persistent exposure define oxidative stress, which is a common feature in all neurodegenerative diseases [49].

The general mechanisms through which As seems to induce oxidative stress are as follows [47,50]:

1. Alteration of the electron transport chain in mitochondria, through inhibition of mitochondrial complex II and IV, determining ROS production and reducing ATP production;
2. ROS production through activation of NADPH oxidase (NOX2);
3. Generation of superoxide anions through reaction of dimethyl-arsine, a metabolite of DMA, with molecular oxygen:
    (CH_3_)_2_AsH + O_2_ → (CH_3_)_2_As• + O_2_^-^•.
    The dimethylarsinic radical can interact with a second molecule of oxygen to produce DMA peroxyl radical:
    (CH_3_)_2_As• + O_2_ → (CH_3_)2AsOO•;
4. Stimulation of the iron release from ferritin that, after further reduction of Fe^3+^ to Fe^2+^, promotes H_2_O_2_ formation through Fenton reaction:
    Fe^II^ + H_2_O_2_ → Fe^III^ + OH• + OH¯;
    FE reduction may be exerted by a human Fe-reductase as Dcytb or, in AD, promoted by Aβ (see paragraph ‘Arsenic and ferroptosis in AD’)
5. Generation of H_2_O_2_ during the oxidation from As^III^ to As^V^:
    H_3_AsO_3_ + H_2_O + O_2_ → H_3_AsO_4_
6. Trivalent As species can bind to thiols contained in proteins determining their misfolding in endoplasmic reticulum. Ultimately, the accumulation of misfolded protein in this subcellular compartment contributes to oxidative species formation;
7. Depletion of the cellular antioxidant pool.

In AD, oxidative stress is a prodromic event that participates in membrane damage, cytoskeleton alterations, and cell death [51].

Reduced glutathione (GSH) is the most abundant non-enzymatic antioxidant in the brain. Structurally, GSH is a tripeptide, in which the reactive part consists in the thiol group contained in a cysteine. GSH antioxidant activity is mediated by some enzymes, of which the most notable are the glutathione peroxidases (GPXs) and catalase (CAT), both involved in the detoxification of H_2_O_2_. The byproduct of these reactions is the oxidized form of glutathione (GSSG), which can be restored to GSH through the activity of glutathione reductase (GR) [52]. Glutathione-S-transferases mediate the addition of GSH molecules to xenobiotics, including As [53].

As exposure can determine a reduction of GSH/GSSG levels, through several mechanisms:

1. Oxidation of trivalent forms of As to their pentavalent analogs;
2. Direct GR inhibition;
3. Augmentation of ROS species;
4. Formation of As-GSH conjugated forms, which facilitates As cellular efflux [34].

A relationship between Aβ and oxidative stress was demonstrated. Specifically, oxidative stress seems to promote Aβ formation, but Aβ itself contributes to oxidative stress [51]. Fewer, but consistent, evidence demonstrated the same interaction for oxidative stress and hyperphosphorylated tau [54].

Hence, in the AD context, As effects on amyloidogenesis and tau hyperphosphorylation, along with the induction of oxidative stress, can contribute to reinforce the vicious cycle responsible for neurodegeneration.

### Arsenic and ferroptosis in AD

Ferroptosis is an iron-dependent form of cell death, in which oxidative stress has a central role. Recent studies have shown that ferroptosis is closely related to the pathophysiological processes of many diseases, including AD [53]. Many compounds with the potential to induce ferroptosis have been developed in recent times. Most of them induce GSH depletion targeting two enzymes involved, respectively, in GSH synthesis and utilization: Xc^−^ and glutathione peroxidase 4 (GPX4). System Xc^−^ is an amino acid antiporter that stimulates the exchange of cystine and glutamate at a 1:1 ratio. Cysteine derived from cystine represents a limiting factor for the synthesis of GSH. GPX4 is a member of the GPX system [55,56]. Iron deposition in the brain of AD patients is observed in the cortex, hippocampus, and basal ganglia. Iron metabolism dysregulation in AD seems to undergo a detrimental cycle in which Aβ down-regulates ferroportin expression, implicating an increase in the labile iron pool. Iron, in turn, reduces the expression of furin, an enzyme that stimulates amyloidogenic processing of APP by increasing BACE1 activity. Finally, Aβ has the ability to reduce iron from Fe^3+^ to Fe^2+^, i.e., the valence state that participates in oxidative species formation through the Fenton reaction. Moreover, hyperphosphorylation of tau protein causes its loss of function in stabilizing APP interaction with ferroportin, impeding iron excretion [46].

To date, growing evidence is unraveling the effect of As on inducing ferroptosis in the brain. In particular, it has been demonstrated that As down-regulates both GPX4 and Xc^−^ expression in the brain of several animal models and cell lines. Consequently, As induces a decrease in GSH content, as well as other antioxidants, including SOD (SuperOxide Dismutase) and CAT. Antioxidant depletion results in increased oxidative stress, as assessed through the analysis of the levels of malondialdehyde, a byproduct of peroxidized lipids. Furthermore, As administration determined the alteration in the expression of iron metabolism-related factors, including ferroportin1, divalent metal transporter1, and transferrin receptor1 [57–59]. Coherently, Xiao et al. demonstrated that treatment with ferrostatin-1, a well-known ferroptosis inhibitor, is able to reverse ferroptotic features induced by As treatment [60].

### Arsenic and DNA methylation in AD

DNA methylation is a process in which DNA methyltransferases (DNMT) transfer a methyl group from a S-adenosylmethionine (SAM) molecule to the 5′ carbon of a cytosine in the DNA to form a 5-methylcytosine (5mC), thereby modulating gene transcription [61]. Instead, the active demethylation process is dependent on the activity of ten-eleven translocation (TET) methylcytosine dioxygenases, which convert 5mC to hydroxymethylcytosines (5hmC), as the primary event of the process [62].

Alterations in DNA methylation in the AD context have been the subject of study for years, and they seem to have a central role in the development of the pathology [63].

In the context of the brain, Yan et al. demonstrated that exposing mice to 5 ppm of arsenic trioxide (ATO) induced significant DNA hypomethylation and altered gene expression in the hippocampus, particularly in genes related to cognition. Moreover, metformin treatment, restoring SAM, partially reverted ATO effects on cognition-related gene expression [64]. Furthermore, Du et al. [65], treating mice with 200 mM of ATO, found a decrease in 5mC and 5hmC in cortex and hippocampus, as well as a decreased expression in all DNMT and TET enzymes, but SAM levels in both the brain regions were conserved. Additionally, Lv et al. [66] reported that 15 mg/l sodium arsenite administration in mice is able to induce a decrease in the general level of 5hmC.

One hypothesis explaining the hypomethylation after As exposure regards SAM depletion due to As metabolism in the body. In fact, SAM acts as a methyl donor for mono- or dimethylation catalyzed by AS3MT, which is also expressed in the brain [67].

Nonetheless, Ríos et al. demonstrated that rats exposed to 3 ppm of sodium arsenite depleted SAM in the liver, but not in the brain [68]. It is possible that higher or more prolonged exposure to As can have a deeper impact on SAM depletion in the brain. Furthermore, this effect can be amplified in the case of a deficiency of those nutrients required to synthesize SAM. SAM is generated through one-carbon metabolism, and dietary deficiency can regard vitamin B6, B9, B12, or methionine [69].

It was also proved that As affects DNA methylation directly regulating the activity of factors involved in DNA methylation, such as DNMTs and TETs [70–72]. As can interact with DNMTs and TETs by bonding with the cytosines contained in the zinc finger domain. As can bind both DNMT3a and DNMT3b, and TETs, but not DNMT1. The nature of the modulation of DNMT3a activity resides in its induction to degradation through the ubiquitin-proteasome system [64,73]. Besides, there is a contradiction regarding the effect of As on the expression of DNMTs and TETs [64,65,74].

Although the cited works agree on As-induced general hypomethylation in the brain, no investigations to date have specifically addressed whether this epigenetic alteration involves AD-related genes, including those already known to undergo hypomethylation in the context of AD [75]. Moreover, evidence collected in different contexts reports both hypo- or hypermethylation after As exposure [76], meaning that more complicated mechanisms can mediate epigenetic effects of As exposure.

Finally, As can also modulate gene expression by DNA methylation or hydroxymethylation through a more general and known mechanism, common to virtually all the oxidant species, directly associating oxidative stress to methylation status, and largely discussed elsewhere [77–79].

## Conclusions

**Figure 1:**
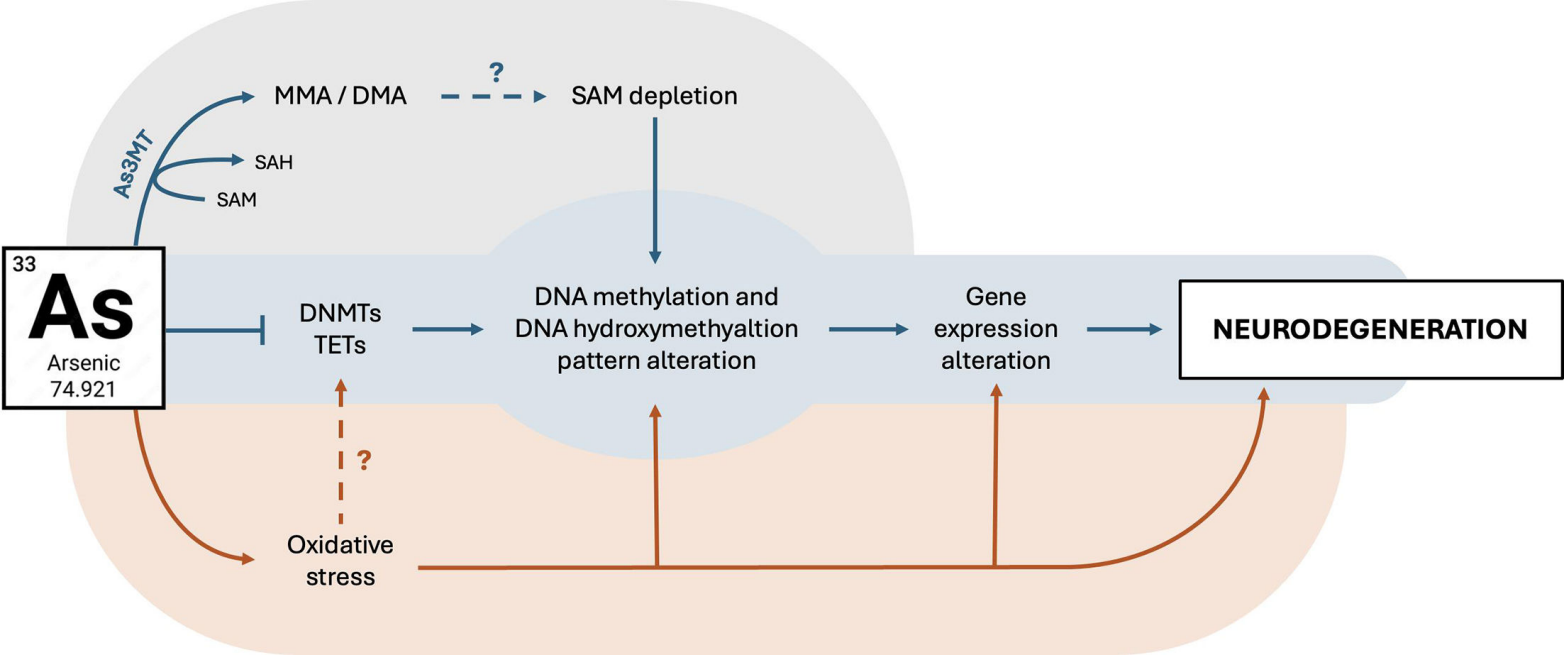
Arsenic and DNA methylation. Arsenic (As) can induce neurodegeneration through several mechanisms, one of which is the alteration of the DNA methylation pattern. It is hypothesized that As metabolism could deplete SAM levels in the brain, although further evidence is needed. Moreover, As inhibits DNMTs and TETs expression or activity. Independently of the underlying mechanisms, alterations of DNA methylation and hydroxymethylation patterns are supposed to determine modulation of gene expression potentially associated with the neurodegenerative process. DNA methylation/hydroxymethylation-associated altered gene expression is also directly induced by oxidative stress. Abbreviations: As, arsenic; As3MT, arsenic(III)-methyltransferase; DMA, dimethylarsenic species; DNMT, DNA methyltransferase; MMA, monometylarsenic species; SAH, S-adenosylhomocysteine; SAM, S-adenosylmethione; TET, ten-eleven translocation methylcytosine dioxygenases.

This review highlights that As exposure represents a significant environmental risk factor in the pathogenesis of AD. Due to its ability to disrupt the BBB, As can accumulate in the brain and contribute to AD development through multiple mechanisms. Specifically, As promotes amyloidogenesis and the formation of neurofibrillary tangles. As one of the few heavy metals that can be metabolized, As metabolism relies on GSH and SAM; their depletion was hypothesized to lead to oxidative stress and, consequently, to epigenetic alterations (Figure 1). However, no definitive experimental evidence supports this assumption; therefore, it would be advisable that further studies would analyze As metabolism and the associated neurodegenerative effects in contexts of altered antioxidant and/or methylation potential. Following As exposure, the brain’s redox balance is disrupted, resulting in oxidative stress and promoting ferroptosis, a specific mechanism of cell death that appears to be relevant to AD. Moreover, several works revealed that As decreases DNA methylation in the brain and interferes with the DNA methylation process, possibly altering gene expression and contributing to cognitive decline. A possible explanation of this effect resides in its ability to interfere with DNMT and TET enzymes and/or in the interaction with SAM levels. Poor evidence directly connects the impact of As on DNA methylation to AD, but the causal effect of DNA methylation has already been demonstrated in the past decades. Therefore, this review, by aggregating knowledge, shows the importance of deepening this aspect. These findings support the possibility of a causal relationship between As exposure and the onset of AD, although further studies are needed to elucidate the molecular mechanisms underlying As-induced neurotoxicity and to develop targeted therapeutic strategies aimed at mitigating its contribution to AD pathology.

**Summary Points**

- Arsenic is associated with Alzheimer’s disease onset.
- Arsenic effects are mediated by mechanisms at the crossroad between oxidation and methylation metabolisms.
- Arsenic metabolism under conditions of impaired SAM availability may generate more toxic species.
- Understanding the toxic pathways associated with metals exposure is helpful to figure out protective strategies.
- Arsenic exposure induces a decrease in DNA methylation in the brain, interfering with DNA methyltransferases and ten-eleven translocation enzymes.

## Open Access

This article has been published open access under our Subscribe to Open programme, made possible through the support of our subscribing institutions, learn more here: https://portlandpress.com/pages/open_access_options_and_prices#conditional

## Abbreviations

AD: Alzheimer’s Disease
APP: amyloid precursor protein
ATO: arsenic trioxide
As: Arsenic
As3MT: Arsenic(III)-methyltransferase
Aβ: amyloid β
BACE1: Beta amyloid cleaving enzyme
BBB: blood brain barrier
CAT: catalase
CDK: cyclin dependent kinase
DMA: dimethylarsenic species
DNMT: DNA methyltransferase
ERK: Extracellular signal-regulated kinase
Fe: Iron
GPX4: glutathione peroxidase 4
GPXs: glutathione peroxidase
GR: glutathione reductase
GSH: glutathione (reduced)
GSK: Glycogen synthase kinase
GSSG: glutathione (oxidised)
MARK: microtubule affinity-regulating kinase
MMA: Monometylarsenic species
MMP: metalloprotease
PSEN1: Presenilin 1
ROS: reactive oxygen species
SAPK: Stress-Activated Protein Kinases
TET: Ten-eleven translocation
WHO: World Health Organization
5hmC: 5mC to hydroxymethylcytosines
5mC: 5-methylcytoine
